# Impact of Flavonoids on Matrix Metalloproteinase Secretion and Invadopodia Formation in Highly Invasive A431-III Cancer Cells

**DOI:** 10.1371/journal.pone.0071903

**Published:** 2013-08-21

**Authors:** Yo-Chuen Lin, Pei-Hsun Tsai, Chun-Yu Lin, Chia-Hsiung Cheng, Tsung-Han Lin, Kevin P. H. Lee, Kai-Yun Huang, Shih-Hsun Chen, Jiuan-Jiuan Hwang, Chithan C. Kandaswami, Ming-Ting Lee

**Affiliations:** 1 Institute of Biochemical Sciences, National Taiwan University, Taipei, Taiwan; 2 Department of Biochemistry, College of Medicine, Taipei Medical University, Taipei, Taiwan; 3 School of Medicine, Taipei Medical University, Taipei, Taiwan; 4 Institute of Biological Chemistry, Academia Sinica, Taipei, Taiwan; 5 Department of Chemical and Biomolecular Engineering, Johns Hopkins University, Baltimore, Maryland, United States of America; 6 Institute of Physiology, National Yang-Ming University, Taipei, Taiwan; 7 Castle Hills Health, Lewisville, Texas, United States of America; University of Bergen, Norway

## Abstract

Metastasis is a major cause of mortality in cancer patients. Invadopodia are considered to be crucial structures that allow cancer cells to penetrate across the extracellular matrix (ECM) by using matrix metalloproteinases (MMPs). Previously, we isolated a highly invasive A431-III subline from parental A431 cells by Boyden chamber assay. The A431-III cells possess higher invasive and migratory abilities, elevated levels of MMP-9 and an enhanced epithelial-mesenchymal transition (EMT) phenotype. In this study, we discovered that A431-III cells had an increased potential to form invadopodia and an improved capacity to degrade ECM compared with the original A431 cells. We also observed enhanced phosphorylation levels of cortactin and Src in A431-III cells; these phosphorylated proteins have been reported to be the main regulators of invadopodia formation. Flavonoids, almost ubiquitously distributed in food plants and plant food products, have been documented to exhibit anti-tumor properties. Therefore, it was of much interest to explore the effects of flavonoid antioxidants on the metastatic activity of A431-III cells. Exposure of A431-III cells to two potent dietary flavonoids, namely luteolin (Lu) and quercetin (Qu), caused inhibition of invadopodia formation and decrement in ECM degradation. We conclude that Lu and Qu attenuate the phosphorylation of cortactin and Src in A431-III cells. As a consequence, there ensues a disruption of invadopodia generation and the suppression of MMP secretion. These changes, in concert, bring about a reduction in metastasis.

## Introduction

Metastasis, the spreading of cancer from the place of origin to distal tissues in the body, is considered to be the principal contributor for mortality in a majority of cancers [1]. Over 90% of cancer-associated deaths are owing to metastasis and yet the mechanisms controlling metastasis remain to be further elucidated [2]. MMPs are the best documented critical proteolytic enzymes that are associated with tumor metastasis [3]. To facilitate metastasis, tumor cells depend on the activity of more than one MMP and of other more general degrading enzymes in order to enable them to cross the tissue barriers they encounter during the process of invasion. It is believed that MMPs degrade the ECM, and that this action enables tumor cells to migrate, invade and spread to various secondary sites in the body where they form metastases. MMPs regulate the tumor microenvironment, and their expression and activation are increased in almost all human cancers when compared with those in the normal tissue equivalent to the cancer [4]. A wealth of recently accrued information suggests that MMPs are recruited to unique surface structures, which are termed invadopodia, and that they are then able to undergo secretion [5], [6].

Invadopodia were first discovered in fibroblasts transformed by the v-src oncogene, which encodes a constitutively active non-receptor tyrosine kinase v-Src [7], and the term was coined in 1989 [8]. Invadopodia are specialized actin-based membrane protrusions found in cancer cells that degrade the ECM via localization of proteases [9]. Their capacity to mediate ECM degradation suggests a critical role for invadopodia in cancer invasion and metastasis. Invadopodia consist of many actin regulatory proteins such as cortactin, N-WASP, Arp2/3 and cofilin [10]. Although these actin regulatory proteins are also components of other actin-based membrane protrusions, matrix-degrading ability stands out as a major, essential and predictable index of invadopodia identification. Three MMPs, MMP-2, MMP-9 and MT1-MMP [11], are reported to be recruited to invadopodia in order to bring about their degrading ability. However, the detailed mechanisms by which MMPs are transported and targeted to the invadopodia, and further secreted remain largely unknown.

In recent years, several molecular players operative in invadopodia have been defined by mutational studies, RNA interference investigations or by the deployment of inhibitory antibodies. Among these, the central role for the Src non-receptor tyrosine kinase in invadopodia regulation has been inferred; subsequent studies have shown that Src kinase activity is essential for invadopodia formation and functioning [12], [13]. Indeed this activity of Src kinase in itself led to the discovery of invadopodia. Phosphorylation of its component proteins is critical for invadopodia regulation and this mainly involves tyrosine phosphorylation by Src kinase. A number of Src substrates, such as cortactin, Tks5, dynamin2, AMAP1/ASAP1 and N-WASP, have been localized to the invadopodia and are required by them to be active [10], [14], [15]. Once Src kinase is activated by upstream integrin signaling or growth factor stimulation, these proteins undergo tyrosine phosphorylation; this then regulates their function as adaptors or allows them to interact with each other during the assembly of the actin networks [12], [16]. Mutation of the tyrosine phosphorylation sites on these proteins inhibits invadopodia formation and functioning [15], [17], which further supports the importance of tyrosine phosphorylation to invadopodia formation. In addition to tyrosine phosphorylation by Src, protein kinase C has also been reported to synergize with Src and to participate in the regulation of invadopodia by phosphorylating serine or threonine [16].

Cortactin, an actin regulatory protein that functions during both the activation and stabilization steps of actin branching, has been recently drawing prominent attention because of its role in invadopodia formation [18]. Cortactin was initially identified as a Src tyrosine kinase substrate [19]. It was named cortactin because it is localized to cortical actin structures. Human cortactin is encoded by *CTTN* (formerly *EMS1*) on chromosome 11q13, which is often amplified in various cancers, such as breast, head and neck [20]. Gene amplification that results in an overexpression of cortactin has been found to be associated with higher metastasis/invasion and a poor prognosis [21]. Consistent with its actin binding ability, cortactin has been found to localize to peripheral cell structures, such as lamellipodia and invadopodia. Recent studies aimed at exploring the molecular mechanisms that regulate actin polymerization prior to MMP recruitment have suggested that cortactin phosphorylation is crucial to invadopodia formation and maturation [22].These findings suggest that Src tyrosine kinase and its substrate cortactin together play highly significant roles in cancer invasion and migration [23].

To determine how cells control invadopodia formation, several investigators have screened a collection of pharmacologically active compounds with the aim of identifying chemicals that are able to inhibit the process. Plant flavonoids have been recognized for some time as possessing anti-tumor and anti-differentiation effects[24]–[27]. Previously, we identified two dietary flavonoid constituents, Lu, a flavone, and Qu, a flavonol, as some of the most potent plant flavonoids in terms of their *in vitro* biological activities. They exhibit a variety of anticancer effects, such as the attenuation of cell growth and kinase activities, the induction of apoptosis, the impairment of differentiation, the suppression of MMP secretion, the reduction in tumor cell adhesion, the inhibition of metastasis and the decrease in angiogenesis[28]–[31]. Although these two flavonoids are potentially effective as anti-invasive compounds, to date no study has assessed the influence of Lu and Qu on invadopodia formation and functioning. In earlier studies, we have documented that both Lu and Qu are able to blunt tyrosine kinase activities and greatly depress the secretion of MMPs in tumor cells [26]. Appreciation of the relevance and importance of the inhibition of kinase activities and of MMP secretion by these flavonoids has prompted us to evaluate their impact on events surrounding invadopodia formation and functioning.

## Materials and Methods

### Materials

A431 human epidermal cancer cell line was purchased from American Type Culture Collection (ATCC) (Manassas, VA). The highly invasive A431-III cells were isolated in our laboratory from the parental A431 tumor cells (A431-P) [32]. Fetal bovine serum (FBS) and RPMI-1640 were obtained from GIBCO (Grand Island, NY). MMP-9 siRNA was purchased from Invitrogen (Carlsbad, CA). PCR primers were purchased from Purigo Biotech (Taipei, Taiwan). Quercetin was purchased from Nacalai Tesque (Kyoto, Japan). Luteolin was obtained from Extrasynthese (Genay, France). Anti-cortactin antibody was obtained from Epitomic (Burlingame, CA). Anti-phospho-cortactin (Y421) antibody was purchased from Cell Signaling Technology (Danvers, MA). Anti-Src antibody was acquired from Upstate Biotechnology (Lake Placid, NY). Anti-phospho-Src (Y418) antibody was purchased from Abcam (Cambridge, MA). Anti-Tks5 antibody and GM6001 were obtained from Millipore (Billarica, MA). Anti-MT1-MMP, TIMP1, TIMP2 antibodies were purchased from Genetex (Irvine, CA).

### Preparation of Cell Lysates

Cells were grown to 80% confluence and then washed with PBS. The cells were lysed with gold lysis buffer as previously described [26]. Insoluble material was collected by centrifugation at 14,000×*g* for 20 min at 4°C. The protein concentration quantified using the Bio-Rad protein assay (Hercules, CA). The samples were then divided into 50 µL aliquots and stored at −80°C for further study.

### Transfection of Small Interference RNA

A431-III cells (2.5×10^5^) were plated into 60 mm culture dishes and allowed to adhere overnight. 6 µl of Lipofectamine 2000 (Invitrogen) was added to 300 µl of serum-free medium, thoroughly mixed and incubated for 5 min at room temperature. In parallel, 12 µl of siRNA stock (10 µM) was added to separate 300 µl of serum-free medium, mixed thoroughly. The diluted siRNA and Lipofectamine 2000 were then combined and incubated for 20 min at room temperature. Finally, the siRNA/Lipofectamine complex was added to the 60 mm dish containing 2.4 ml serum free medium giving a final concentration of 40 nM. After 24 h the medium containing the transfection complex was changed and fresh medium containing 10% FBS was added; the cells were then incubated for another 24 h. All assays were performed 48 h after transfection.

### Quantitative Real Time PCR

Total RNA was isolated by using High Pure RNA Isolation Kit (Roche, Basel, Switzerland), and reverse transcribed by using the MMLV High Performance Reverse Transcriptase kit (Epicentre, Madison, WI). Quantification of the transcript levels of target genes was performed in the LightCycler system (Roche) using a commercial SYBR Premix Ex Taq (Takara Bio, Shiga, Japan) and specific primer sets.

### Gene Expression Microarray Analysis

The procedure of gene expression microarray analysis was provided by manufacturer Welgene Biotech (Taipei, Taiwan). Briefly, both A431-P and A431-III cells (1×10^6^) were plated onto 100-mm dishes and allowed to grow in complete medium. After 24 h, the totals RNA of both cells were extracted by 3 mL Trizol reagent. The gene expression microarray analysis of A431-P and A431-III cells were performed by Welgene Biotech. The data discussed in this publication have been deposited in NCBI’s Gene Expression Omnibus and are accessible through GEO Series accession number GSE47996 (http://www.ncbi.nlm.nih.gov/geo/query/acc.cgi?acc=GSE47996).

### Gelatin Zymography

The conditioned media was collected and separated by 8% SDS-PAGE containing 0.1% gelatin. After electrophoresis, the gel was washed in 2.5% Triton X-100 for 20 min, twice, to renature the gelatinases and then incubated in reaction buffer (50 mM Tris–HCl, pH 8.0, containing 5 mM CaCl_2_, 0.02% NaN_3_) at 37°C for 24 to 48 h. The gel was then stained with Coomassie Blue and destained with destaining buffer as previously described [33]. The gelatinase activity was visible as clear zones within the gel.

### Western Blotting

The cell lysate samples were mixed with 5× sample buffer and boiled for 5 min, separated on 10% SDS-polyacrylamide gels (PAGE), and then transferred to nitrocellulose membrane (Millipore). The membrane blots were blocked in PBS containing 5% BSA for 1 h at room temperature, and incubated with primary antibody overnight at 4°C. After washing with TBST containing 20 mM Tris-HCl (pH 7.6), 0.8% (w/v) NaCl and 0.25% Tween-20, the blots were incubated with secondary antibody conjugated with horseradish peroxidase (Millipore). The membranes were then washed with TBST, and immunoreacted bands were detected with ECL reagents and exposed on Fujifilm.

### In Vitro Invasion Assay

The filter of a 24-well Transwell unit was coated with 0.1 mL of 0.6 mg/mL EHS Matrigel. The lower compartment contained RPMI-1640 with 10% FCS as a chemoattractant. The cells were placed in the upper compartment (10^5^ cells/0.5 mL RPMI-1640 containing 0.1% BSA) for 24 h. After incubation, the filters were fixed with 3% glutaraldehyde in PBS and stained with crystal violet. Cells on the upper surface of the filter were gently scraped off, and those that penetrated through the Matrigel to the lower surface of the filter were counted under a microscope (20×).

### Immunofluorescence

Cells were plated on 6-well plate containing 18 mm gelatin-coated coverslips for 18–24 h in order to adhere. The medium was then discarded and the cells were fixed with 4% paraformaldehyde for 15 min, washed with PBS, and permeabilized with 0.1% Triton X-100 for 10 min. After washing with PBS, the coverslips were blocked with 1% BSA in PBS for 30 min. The cells were then incubated with anti-cortactin or anti-Tks5 antibodies, which were diluted in 1% BSA blocking buffer for 1 h. This was followed by incubation with the appropriate secondary antibody conjugated to Alexa fluro 555 (Invitrogen) for 1.5 h. The same cells were also stained with Alexa fluro 488-conjugated phalloidin and DAPI to detect F-actin and the cell nuclei, respectively. The coverslips were then mounted in 50% glycerol in PBS and analyzed by confocol image microscopy.

### Matrix Degradation Assay

The matrix degradation assay was performed according to a previously described procedure [34], and the whole procedure for the matrix degradation assay was performed in the dark. In brief, 18 mm coverslips were first coated with 0.2 mg/mL Oregon Green® 488-conjugated gelatin (Molecular Probe). The coverslips had 200 µL of 0.5% ice-cold glutaraldehyde in PBS added onto them and the mixtures was then incubated for 15 min at 4°C. After washing with PBS, the coverslips were incubated with fresh 5 mg/ml NaBH_4_ in PBS for 3 min at room temperature. Next, the coverslips were washed with PBS and sterilized in 70% ethanol. After quenching with serum-free medium for 1 h, 1×10^5^ cells were seeded on coverslips for 5 h, and slides were processed for immunofluorescence analysis using phalloidin to detect F-actin and DAPI to detect the cell nuclei. The images were visualized by confocal microscopy. To quantify invadopodia formation and function, the invadopodia were manually quantified by counting the total number of cells producing invadopodia in at least five individual fields (>300 cells). The area of matrix round the cells that had been degraded was also measured using an identical signal threshold for the Oregon Green® 488-gelatin fluorescence for every image. The degraded area measured was the area where the fluorescence signal was below the threshold as measured by ImageJ. The quantified area was then normalized against the number of cells.

### Confocal Microscopy

Fluorescent images were captured using a Zeiss LSM510 confocal microscope with a 63× oil immersion lens, a NA 1.25 objective and pin hole of 0.1 airy unit. Image analysis was performed using ImageJ software.

### Statistical Analysis

Quantitative data from at least three independent experiments are expressed as means (±SEM). Unpaired Student’s t-tests were used to compare the differences between groups. A *p* of <0.05 was considered statistically significant.

## Results

### A431-III Cells Form more Invadopodia and Degrade the ECM Effectively

**Figure 1:**
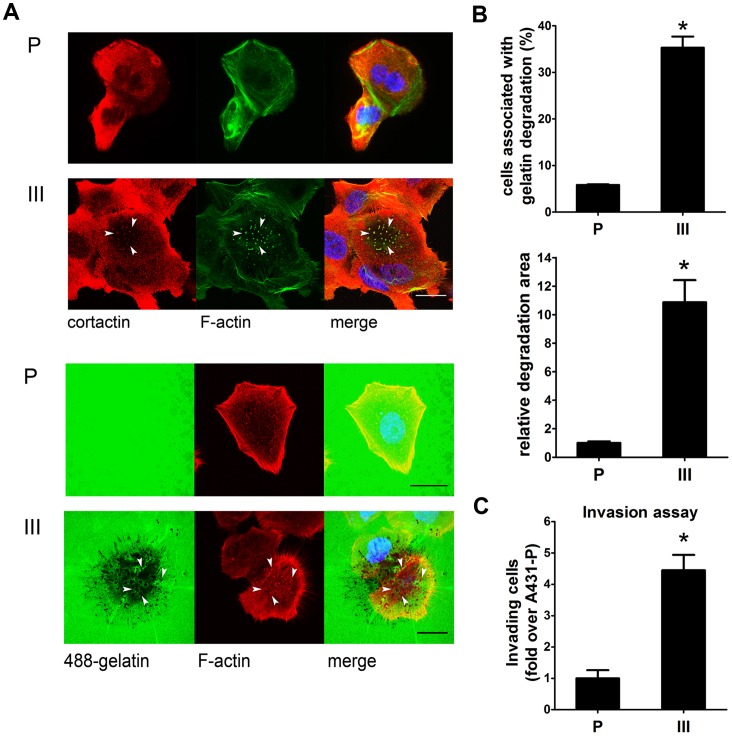
A431-III forms invadopodia and exhibits higher ability to degrade gelatin than A431-P. A, Upper panel: A431-P and A431-III cells were stained with cortactin (red), F-actin (green), and DAPI (blue). Arrowheads, examples of invadopodia that are identified as cortactin and actin-positive dots. Representative images taken of both cells. Lower panel: Both cells were plated on Oregon Green® 488-conjugated gelatin. Degraded ECM was identified as a dark area on the gelatin. B, Upper panel: Quantification of cells associated with matrix degradation. Lower panel: Quantification of the degradation area normalized against cell number. C, Invasion assays were performed. *p<0.05. Error bars present the standard error of the mean. Scale bar are 22 µm. P (A431-P); III (A431-III).

We have previously shown that the A431-III cells exhibit greater invasive and migratory capacities together with elevated levels of MMP-9 [32]. This provides us a reliable model for studying the mechanism of metastasis/invasion by comparing A431-III cells with the parental cells (A431-P). To determine whether A431-P and A431-III cells are able to form invadopodia, we first performed immunofluorescence to detect and compare the formation of invadopodia by A431-P and A431-III cells. A431-P and A431-III cells were plated on gelatin coated 6-well plates for 4 h. Invadopodia could be observed as cortactin and actin-positive dots located on ventral site of cells. As shown in figure 1A, invadopodia were easily found in A431-III cells. There were few or no detectable invadopodia visible in A431-P cells. Next, matrix degradation assay was performed to evaluate the ECM degradation capacity of invadopodia in A431-P and III cells. The A431-III cells were found to have higher gelatin degrading ability compared with A431-P cells (Fig. 1A). This data suggests that the greater gelatin degrading ability found to be associated with A431-III cells parallels their greater ability to form invadopodia.

To quantify the formation of invadopodia and their ability to degrade the ECM, we calculated the percentage of cells with invadopodia puncta that were associated with degraded gelatin patches. As shown in figure 1B, over 35% of A431-III cells were found to form invadopodia and to degrade gelatin. This differs with the results for A431-P, wherein only 5% of A431-P cells were found to form invadopodia. We also quantified the area of the degraded patches and normalized this against cell numbers. A431-III cells exhibited a 10-fold higher ability to degrade gelatin than did A431-P cells. This greater ability of invadopodia formation and degrading function corresponded with the invading ability of these cells (Fig. 1C). In summary, these findings suggest that, when compared to A431-P cells, highly invasive A431-III cells exhibit a much greater ability to form invadopodia and that this leads to more degradation of ECM and increased invasive ability.

### Cortactin Phosphorylation Promotes Invadopodia Formation and Matrix Degradation

**Figure 2:**
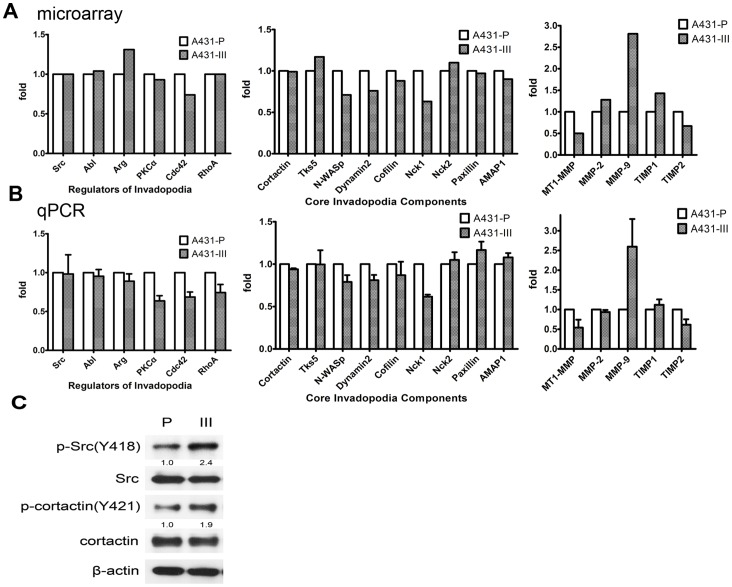
Src kinase activity and phosphorylation of cortactin were responsible for invadopodia formation in A431-III cells. A, Expression of invadopodia regulators, core components and MMPs/TIMPs in A431-P and A431-III were analyzed by microarray. B, Expression of invadopodia regulators, components and MMPs/TIMPs were validated by qPCR. C, Total cell lysates were subjected for immunoblotting analysis. The active status of Src kinase and the phosphorylation of cortactin were determined.

Recently, several reports have approved that the overexpression of invadopodia regulators or invadopodia component proteins are able to enhance invadopodia formation. To further confirm whether these regulators are up-regulated in A431-III cells, we performed microarray and qPCR analyses to elucidate this event. To our surprise, there was no significant elevation of any known invadopodia regulator or component protein (Fig. 2A&2B). This data suggests that the difference between A431-P and A431-III, in terms of invadopodia, is likely to be at signal transduction level rather than at protein expression level.

Src kinase plays a fundamental role in the regulation of invadopodia formation [12], [13]. Several component proteins are known to undergo tyrosine phosphorylation during invadopodia genesis, including cortactin [9], [35]. Phosphorylation of cortactin by Src kinase is a key switch that allows remodeling of actin filaments and the activation of invadopodia formation and functioning [17], [22]. To test the role of Src in invadopodia formation and function, we examined Src kinase activity by anti-phosphorylated Src antibody in A431-P and III cells. As shown in figure 2C, we found that Src kinase was significantly activated in A431-III cells, and followed by an increase in phosphorylation of the downstream target, cortactin. These findings suggest that the Src kinase activity, rather than the transcriptional induction of Src or of any other invadopodia regulator/component, is likely to be responsible for increased invadopodia formation by A431-III cells, in concordance with our inference concerning the microarray data.

**Figure 3:**
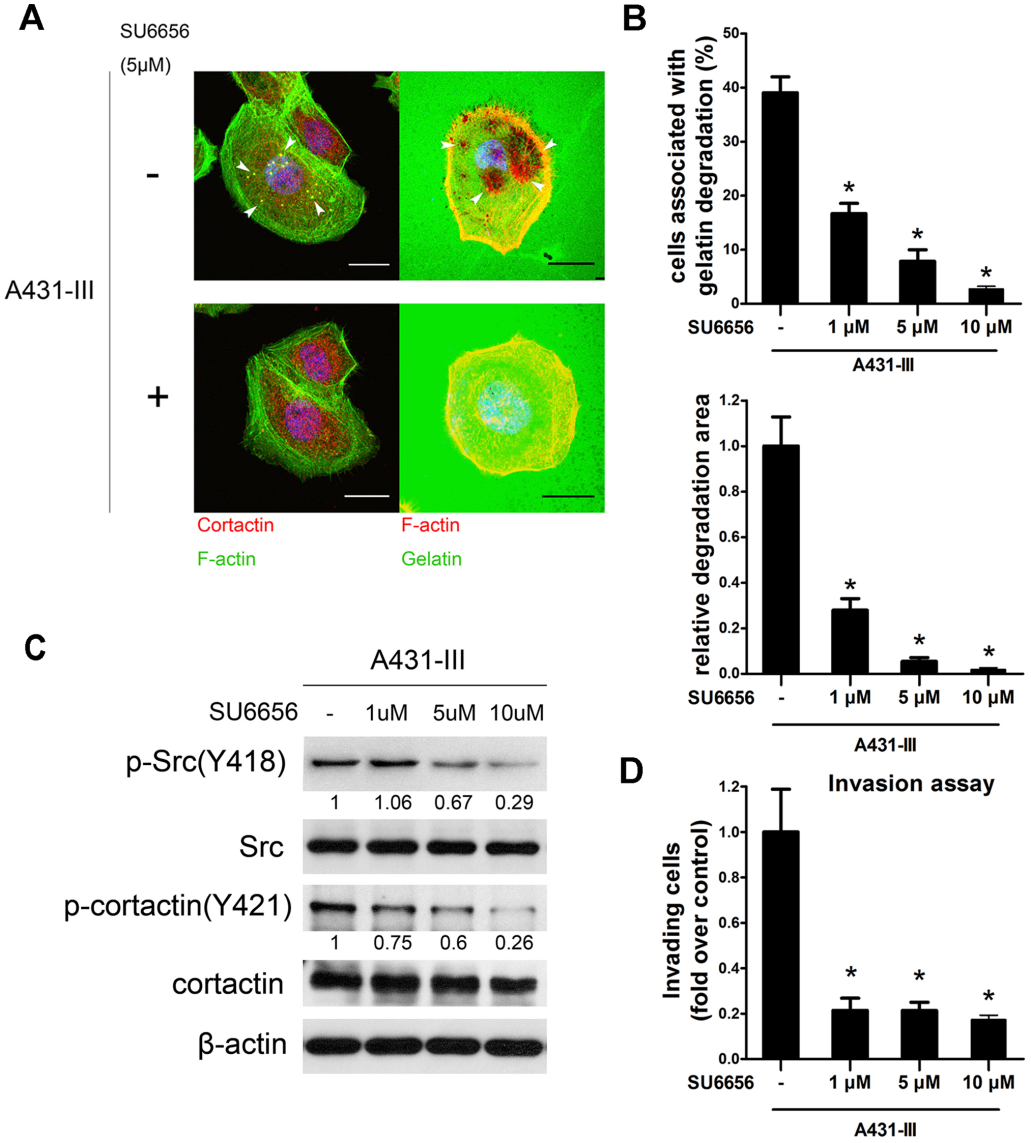
Effects of SU6656 on invadopodia formation and functioning. A, A431-III cells were plated on gelatin or Oregon Green® 488-conjugated gelatin and treated with DMSO or 5 µM SU6656 for 5 h to investigate the formation of invadopodia and matrix degradation. B, Quantification of cells associated with matrix degradation (upper panel). Quantification of the degradation area normalized against cell number (lower panel). C, Total cell lysates were prepared for immunoblotting analysis. Active Src and downstream target cortactin (Y421) were analyzed. D, Invasion assays were performed. *p<0.05. P values are compared with control A431-III. Error bars present the standard error of the mean. Scale bar are 22 µm.

To further confirm the role of Src kinase and cortactin phosphorylation in invadopodia formation in A431-III cells, we then treated cells with SU6656, a selective Src kinase inhibitor. The result showed that invadopodia formation was dramatically suppressed by the treatment with SU6656 (Fig. 3A), as were the invading abilities detected by invasion assays (Fig. 3D). Quantification of the percentage of invadopodia-positive cells and relative degradation area are shown in figure 3B. These findings demonstrate that a reduction in phosphorylation of Src results from the inhibition of Src kinase activity by SU6656, while also accompanied by a lower level of cortactin phosphorylation (Fig. 3C). This data suggests that Src kinase activity is necessary for invadopodia formation and the invasive potential.

### MMP Activity is Required for Invadopodia Formation and Matrix Degradation

**Figure 4:**
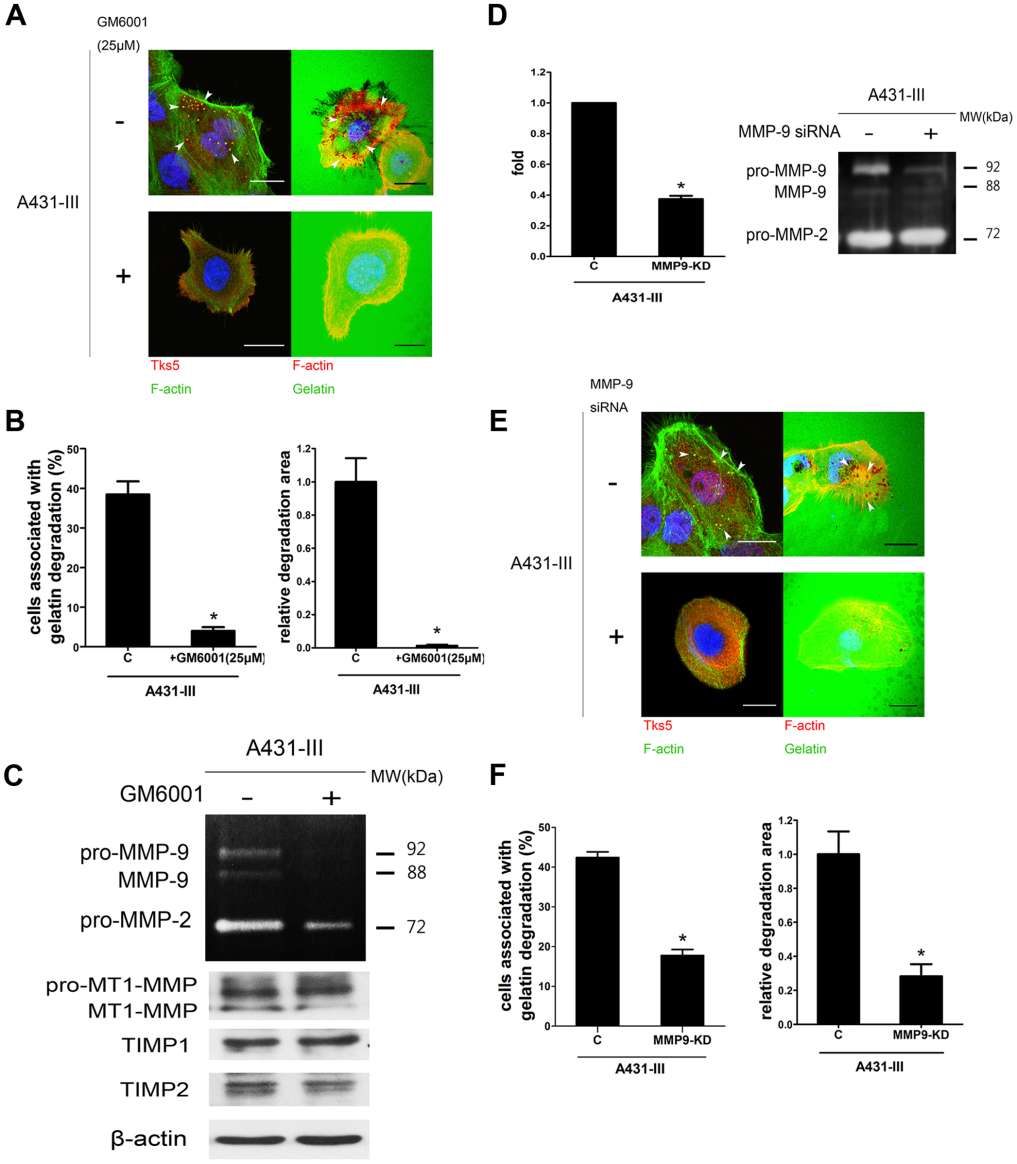
MMPs, especially MMP-9, were responsible for the invadopodia and degrading ability of A431-III cells. A, A431-III cells were plated on gelatin or Oregon Green® 488-conjugated gelatin and treated with DMSO or 25 µM GM6001 for 5 h to observe the formation of invadopodia and the matrix degrading ability. Tks5, invadopodia component protein, was used as a marker. B, Quantification of cells associated with matrix degradation (left panel). Quantification of degradation area normalized against cell number (right panel). C, Effect of GM6001 on MMPs’ activities and TIMPs’ expression were measured by zymography and western blot. D, The cells were treated with 40 nM MMP-9 siRNA or control siRNA. Knockdown efficiency was measured by qPCR (left) or gelatin zymography (right). E, A431-III cells (expressing control or MMP-9 knockdown siRNA) were plated on gelatin or Oregon Green® 488-conjugated gelatin to investigate the formation of invadopodia and the matrix degrading ability. F, Quantification of cells associated with matrix degradation (left panel) and degradation area normalized against cell number (right panel).*p<0.05. Error bars present the standard error of the mean. Scale bar are 22 µm.

To metastasize, tumor cells rely on invadopodia formation and MMPs to accomplish the digestion of the ECM, which facilitates cell penetration across the ECM barrier. The involvement of MMPs in invadopodia activity is essential if proteolysis were to occur. To determine the correlation of MMPs and invadopodia formation in A431-III, we first measured the ability of A431-III cells to form invadopodia and degrade the ECM in the presence of GM6001, a broad spectrum MMP inhibitor. Our study used Tks5 as a marker to detect invadopodia formation and functioning. Treating A431-III cells with 25 µM GM6001 completely abolished gelatin degradation (Fig. 4A). No detectable invadepodia dot-like structures in A431-III cells was observed after treatment with GM6001. This effect might be owing to invadopodia’s failure to form a stable structure when MMP activity is inhibited. Quantification of the percentage of invadopodia-positive cells and of the relative degradation areas are presented in figure 4B. The effects of GM6001 on MMPs’ activities and TIMPs were measured by western blot and zymography (Fig. 4C). Together, our findings show the critical importance of MMP activity in the degrading ability of A431-III cells, and how MMP activity influences the formation of invadopodia structure.

Three MMPs, MT1-MMP (MMP14), MMP-2 and MMP-9, have been reported to be associated with invadopodia functioning in various cell lines [13], [36], [37]. We have shown that MMP-9 activity is the key MMP which markedly increases in A431-III cells (Fig. 2A&2B) [32], and that this protein plays a significant role in migration, invasion and the EMT [33]. Due to the fact that MT1-MMP and MMP-2 undergo little or no changes in A431-III cells, it is thus likely that an elevation in MMP-9 level is the major contributing factor for the increase in invadopodia proteolytic activity. To confirm whether MMP-9 contributes to invadopodia formation process, a blockade of endogenous MMP-9 activity in A431-III cells was carried out by siRNA knockdown. Knockdown of MMP-9 in A431-III cells were confirmed by qPCR and zymography analyses (Fig. 4D). The loss of MMP-9 resulted in a failure to detect invadopodia puncta in A431-III cells (Fig. 4E). Knockdown of MMP-9 in A431-III cells was sufficient to impair invadopodia formation by approximately 60% and cause a reduction of 70% in the total area degraded by these cells as is shown in figure 4F. In general, knockdown of MMP-9 in A431-III cells had a similar result to that of exposure to GM6001. In both cases there was either a failure to detect invadopodia or a significant reduction in the number of invadopodia dot-like structures. Collectively, these findings indicate that MMP-9 plays an important role in the induction of invadopodia generation and the subsequent degradation of the ECM.

### Luteolin and Quercetin Inhibited Src Kinase Activity and Affected the Downstream Target Cortactin

In our previous studies, we identified two of the most potent known flavonoids, namely Lu and Qu. These flavonoids exhibit a variety of anti-cancer effects, such as inhibition of cell growth and kinase activity, suppression of MMPs expression and secretion, decrease in migration/invasion, and reversal of EMT [26], [29], [38], [39]. Although these two flavonoids are potentially effective as anti-invasive compounds, to date no study has assessed the influence of Lu and Qu on invadopodia formation and functioning. Based on their effects on kinase activity and MMPs [26], we postulated that Lu and Qu might have the ability to disrupt the formation and functioning of invadopodia. Suggestions have been made that Src, once activated, is able to transduce signals to downstream targets that then modulate cell growth, survival, migration and invasion [40]. These findings prompted us to explore whether treatment with either Lu or Qu would have a direct inhibitory effect on Src tyrosine kinase activity, and impairment of cortactin phosphorylation as a consequence.

**Figure 5:**
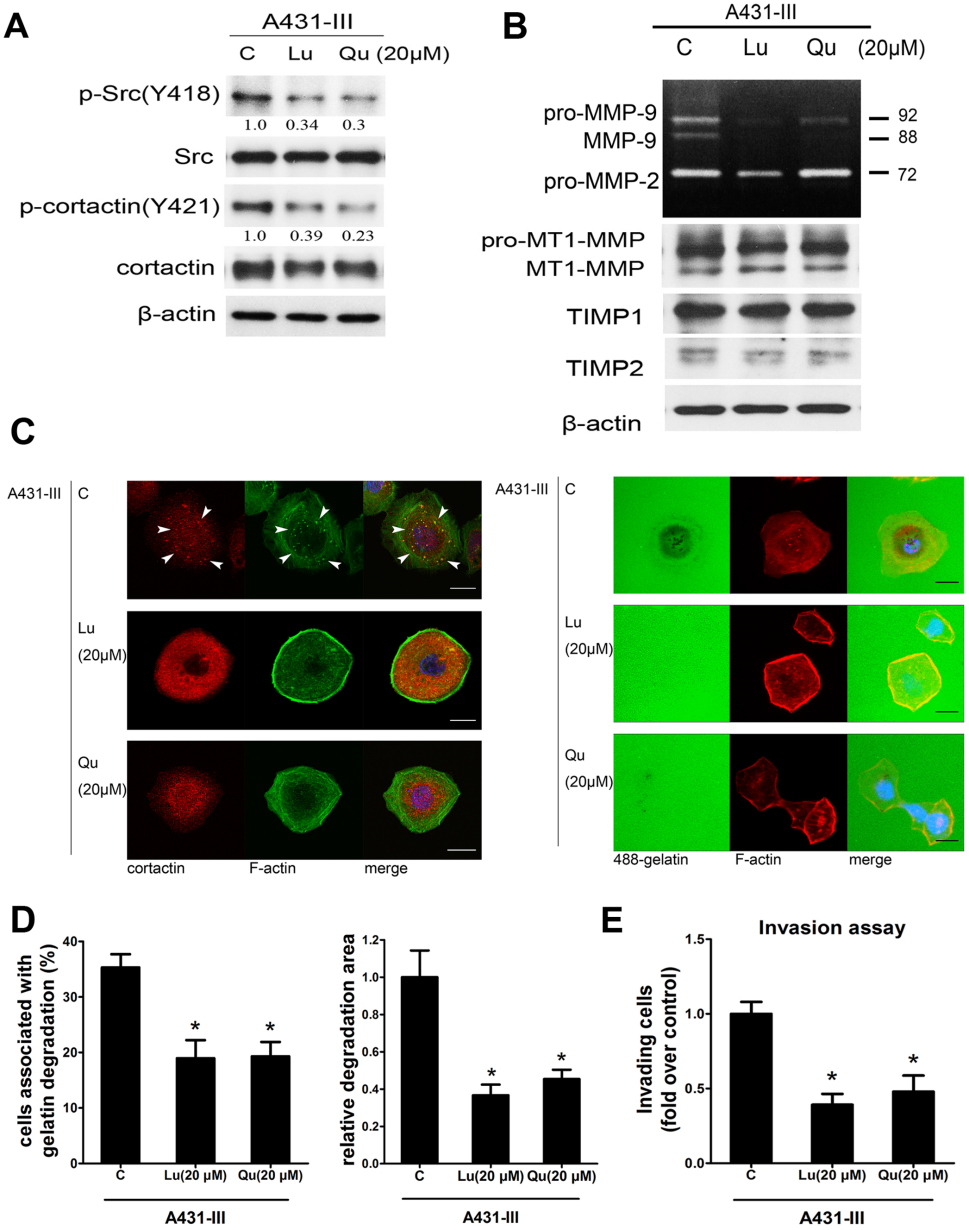
Lu and Qu inhibited Src kinase and MMPs secretion, impaired invadopodia formation and matrix degradation. Cells were treated with 20 µM Lu or Qu in serum free medium for 24 h. A, Total cell lysates were subjected for immunoblotting analysis. Active Src and downstream target cortactin (Y421) were determined. B, Conditioned media and cell lysate were analyzed for MMPs’ activities and TIMPs’ expression using gelatin zymography and western blot. C, Representative images of A431-III treated with Lu or Qu. Left panel: The cells were stained with cortactin (red) and F-actin (green). Right panel: A431-III cells were plated on Oregon Green® 488-conjugated gelatin to investigate the matrix degrading ability. D, Quantification of cells associated with matrix degradation (left panel). Quantification of degradation area normalized against cell number (right panel). E, Invasion assays were performed. *p<0.05. P values are compared with control A431-III. Error bars present the standard error of the mean. Scale bar is 22 µm.

Treatment with either Lu or Qu was found to diminish Src phosphorylation and phosphorylation of cortactin (Fig. 5A). In addition to the inhibitory effect on Src and cortactin phosphorylation, we also investigated the effects of these two flavonoids on MMP activity. As shown in Fig. 5B, Lu or Qu suppressed the secretion of MMP-2 and MMP-9 activity, but had no significant effects on MT1-MMP, TIMP1 and TIMP2.

To further investigate the impact of Lu and Qu treatment on the invadopodia formation process, we evaluated the ability of A431-III cells to form invadopodia and degrade gelatin in the presence of Lu or Qu. As shown in figure 5C, we observed that both Lu and Qu did inhibit the formation of invadopodia and reduce gelatin degradation. Among the untreated control A431-III cells, more than 36% of the cells were found to form invadopodia. On treatment with luteolin or quercetin, the number of invadopodia formed was down to 17% and 18% of all cells, respectively (Fig. 5D). Furthermore, the degradation area was also diminished to 36% and 45% comparing to the control A431-III cells (Fig. 5D). We also performed invasion assays to further confirm that these inhibitions of invadopodia and degrading ability finally caused the decrements of invading ability (Fig. 5E). These findings reveal that Lu and Qu, when used to treat A431-III cells, were able to effectively impair the formation of invadopodia, reduce the ability to degrade gelatin, and finally inhibit the invasion.

## Discussion

We previously documented that A431-III subline displays high levels of MMP-9 secretion accompanied with increased spread, migration and invasion compared to that in A431-P cells [32]. Furthermore, A431-III cells express much greater levels of various EMT markers, including fibronectin, vimentin, Twist and Snail, which are likely to be the cause of E-cadherin being switched to N-cadherin, resulting in the loss of cell-cell junctions [33]. In light of these findings, it seemed likely that a comparison of the tumor progression events evoked in A431-P cells with those in A431-III cells might be a useful strategy to probe MMP secretion via invadopodia. Recently, invadopodia has acquired status as dynamic and crucial structures that are central to proteolytic activity by cells to invade ECM [6]. In this study, we first examined whether invadopodia were formed and functioned in A431-P and A431-III cells. The data showed that A431-III cells were able to form more invadopodia and exhibit a higher matrix degrading ability than that of A431-P cells (Fig.1). The rises in invadopodia generation and the higher level of matrix degradation by A431-III correlate with elevated MMP secretion and a higher invasive potential. Numerous reports have shown the overexpression of regulators and/or component proteins of the invadopodia, including cortactin and Tks5 in highly invasive cancer cells. Such accentuated expression appears to be intimately linked with a poorer patient prognosis.

To elucidate the possible impact of cortactin on invadopodia formation, we next examined whether the expression level of cortactin is correlated with invadopodia formation and matrix degradation. Recently, several reports have provided evidence suggesting that the overexpression of invadopodia regulators or component proteins are able to enhance invadopodia formation. We characterized expressions of regulators and component proteins of invadopodia by RNA microarray analysis and then further analyzed them through qPCR. To our surprise, there was little or no measureable change in the regulators/components of the invadopodia between A431-P and A431-III cells as can be seen in figure 2A&2B. The two exceptions were an about 30% increase in Arg and an about 18% decrease in Cdc42 in A431-III cells. It is worth noting that Cdc42 is necessary for invadopoda formation as it is among the upstream regulators of N-WASP. In this context it is unexpected that Cdc42 expression would decrease in highly invasive A431-III cells. Future studies are warranted in regard to this issue. Notably, microarray analysis revealed only a slight increase in Tks5 and a slight decrease in N-WASP, Nck1 and Dynamin2, which are core invadopodia components in the comparison between A431-III cells with A431-P cells. Our results are at variance with other reports showing the up-regulation of the regulators and components associated with invadopodia in highly invasive tumor cells. It is well recognized that microarray datasets do not accurately represent endogenously produced mRNA levels within cells. Microarray results are rather based on a standard algorithm that is computed from many factors including signal intensity, the number of genes present and the fold changes that occur. Thus, the lack of measureable microarray changes in the regulator and component genes of the invadopodium when A431-III cells are compared with A431-P cells is not unusual.

Due to the fact that Src kinase and cortactin phosphorylation are the master switches for invadopodia formation, and the fact that we did not see significant differences between A431-P and A431-III cells, we examined the phosphorylation status of Src kinase and its downstream cortactin. There was a 2.4-fold increase in p-Src and 1.9 fold increase in p-cortaction for A431-III cells when compared with that for A431-P cells (Fig. 2C). We quickly recognized that active Src kinase and phosphorylation of cortactin might be responsible for invadopodia formation and functioning, rather than just changes in protein expression levels of Src and cortactin. It is worth noting that the A431 cell model system is distinctly different from that of cell types vogue in current studies targeting invadopodia, which mainly employ Src-transformed cells [41] or metastatic cell lines [12], [17], [42], [43]. This A431 system may provide another approach to study the importance of invadopodia and may be used for further studies of molecular mechanism of invadopodia.

SU6656 is a selective Src family kinase inhibitor that was first used to probe growth factor signal pathways [44]. As shown in figure 3, SU6656 greatly inhibits the formation of invadopodia and affects matrix degradation in a dose dependent fashion. SU6656 also dramatically impairs Src kinase activity, thus decreasing the phosphorylation of cortactin (Fig. 3C). Our finding is in concordance with reports suggesting that Src kinase activity is required for both invadopodia formation and functioning [13]. Decreased p-cortactin level leads to a diminution in invadopodia puncta elaboration. These findings suggest a prime role for p-cortactin as a link between several invadopodia formation processes, including Src kinase signaling, actin assembly and MMP trafficking. In addition, p-cortactin might be a key switch controlling invadopodia formation and function. We conclude that recruitment of p-cortactin to invadopodia ensues first, to be followed by MMP accumulation and ECM degradation. The phosphate groups on cortactin might play a role in the recruitment of MMPs and their subsequent binding to the invadopodia structure, rather than as a regulator of actin dynamics. However, this remains to be further evaluated and corroborated.

Recently, many studies have shown that MMPs are enriched at invadopodia, including MMP-2, MMP-9 and MT1-MMP, which are then involved in ECM degradative action [11]. In A431-P and A431-III cells, MMP-2 and MMP-9 are detectable in the medium after 12 h of cell culture, and these proteins increase steadily over time. In this study, treatment of A431-III cells with GM6001 effectively abolished ECM degradation at the invadopodia sites and greatly diminished the number of invadopodia. These findings imply that only active MMPs are concentrated at the invadopodia sites where matrix degradation occurs. Although results obtained using MMP inhibitors appear to be promising in preclinical studies, they have failed to show efficacy in clinical trials [45]. In addition, such inhibitors reportedly display acute toxicity, which compromises their potential therapeutic applications [46]. Invadopodia formation is crucial for pericellular proteolysis and invasion through the ECM. In this study, we implemented a siRNA strategy to reduce MMP-9 release by A431-III cells in order to confirm our conjecture that *MMP-9* is *required* for *invadopodia formation*. It is known that MT1-MMP/MMP-2 are found to localize in invadopodia, and the MT1-MMP/MMP-2 axis is required for the activation of proMMP-9. However, our data indicate that only MMP-9 is up-regulated in A431-III (Fig 2A&2B) [32]. Thus, in this study, we focus on the role of MMP-9 in invadopodia formation and its function. Compared to the control, MMP-9 siRNA greatly reduced the expression of MMP-9 and simultaneously decreased the degradative capacity of A431-III cells (Fig. 4). Considering the data on GM6001 inhibition of MMP-9 activity and siRNA knockdown of MMP-9 expression, both result in a decrement in invadopodia dot-like structure formation. These findings confirm the critical importance of MMP-9 with respect to invadopodia formation.

Previously, we have reported that both Lu and Qu are able to inhibit a wide spectrum of kinase activity and the secretion of MMPs [26]. There are other recent reports supporting our hypothesis that Src kinase is one of the targets for interception by these flavonoids [47]. Based on these results, we speculate that Lu and Qu are likely to be excellent candidate drugs for inhibiting Src kinase activity and should be able to disrupt invadopodia formation and functioning. In the present study, we have also shown that treatment of cell lines with Lu or Qu suppresses the phosphorylation of Src and cortactin (Fig. 5A), which would reduce MMP-9 secretion by A431-III (Fig. 5B). It should be emphasized that neither Lu nor Qu nor MMP-9 siRNA treatment results in a measurable change in Src and cortactin at either the protein (Fig. 5A) or mRNA levels (data not shown). These findings further substantiate the critical role of MMP-9 in the modulation of invadopodia formation and functioning. However, details of the mechanism through which inhibition occurs remains to be elucidated. Our previous study found that secretion of MMP-9 was blocked by Lu and Qu treatment, and that MMP-9 was found to accumulate in the cell lysate [38]. Our study here shows that invadopodia machinery and invadopodia predicated functions are blunted by the two flavonoids. Together, these two studies provide clues that the ability of flavonoids to inhibit MMP secretion might be owing to the inhibition of invadopodia formation. Ammer et al. showed that inhibition of Src kinase results in inhibition of MMP-9′s secretion, causing an accumulation of MMP-9 in HNSCC cell lines [48]. Moreover, MMPs have to be recruited to the invadopodia in order to accomplish the degradation of the matrix. In this context, Clark and Weaver showed that cortactin, which is recognized as an actin regulatory protein, not only is an actin cytoskeletal regulator but also a modifier of the secretion of MMP-2/MMP-9 and an attendant transportation of MT1-MMP to the membrane [18]. The study proposes a novel mechanism directly linking the formation of invadopodia to vesicular trafficking. While it is held that cortactin is connected to the secretion of MMPs, the importance of phosphorylation of cortactin in this process remains fuzzy or not clear, even though the phosphorylation of cortactin is actually the critical switch by which cortactin leads actin network assembling [49]. In follow-up experiments, our results demonstrated that the two flavonoids dramatically affected invadopodia formation (Fig. 5C). We suggest that this phenomenon primarily occurs due to an influence on the phosphorylation levels of cortactin that is mediated by Src, rather than the effect of an abundance of cortactin. Thus, there is a direct connection between the phosphorylation of Src, the phosphorylation of cortactin, and MMP secretion. As alluded to above, we recognize that Src kinase regulates invadopodia formation. Under these circumstances, there is up-regulation of several signaling pathways in A431-III cells and also activation of certain kinases, including Akt and ERK, both of which have higher phosphorylated levels [38], [50]. These proteins reportedly also interact with Src and contribute to a complex network of signal transduction [51]. Assessment of these findings supports our contention that higher quantities of activated Src exists in A431-III cells as compared to that in A431-P cells.

Finally, there is a need to explore EMT in relation to invadopodia. EMT is a biological process in which epithelial cells lose their characteristic polarity and dissemble their cell-cell junctions in order to acquire increased motility; this is currently regarded as a crucial event in the onset of cancer cell migration, invasion and metastasis [52]. On the basis that EMT leads to increased cancer cell motility and invasiveness and that invadopodia are the structures which execute matrix degradation, it must be assumed that the EMT somehow must invoke or prime the formation of invadopodia. Nevertheless, at present there only have been very limited studies that directly link the EMT with the formation of invadopodia. The association between EMT and invadopodia still remains largely unknown and undefined. Though the role of Twist seems clear in invadopodia [53], there is a wealth of transcription factors that are known to participate in the EMT process such as Snail, Slug, ZEB1, and ZEB2; their role in formation and functioning of invadopodia needs elucidation. Moreover, vimentin, an intermediate filament protein that is recognized as EMT marker, allegedly cooperates in the elongation of invadopodia [54]. Previously, we have reported that Snail expression in A431-III cells seems to be induced by the upregulation of MMP-9, resulting in portal invasion and EMT [33]. Since we have established the A431 system that presents with EMT phenotype, we herein revealed the role of invadopodia in this system. This A431 system could be a reliable model for further investigation into the relationship between EMT and invadopodia in cancer invasion.

In summary, our study provides a reliable model to investigate and confirm the importance of invadopodia in cancer invasion. Our findings also demonstrate that both Lu and Qu are able to target invadopodia and prevent cancer cell invasiveness. In general, these two polyphenolic flavonoids not only ablate the EMT process, but also abrogate invadopodia formation. Thus, Lu and Qu appear to have inherent potential as chemotherapeutic agents that would be able to attenuate tumor progression through inhibition of invadopodia formation.
